# A pilot study of allostatic load among elderly Japanese living on Hizen-Oshima Island

**DOI:** 10.1186/1880-6805-31-18

**Published:** 2012-06-25

**Authors:** Douglas E Crews, Hajime Harada, Kiyoshi Aoyagi, Takahiro Maeda, Alexandria Alfarano, Yoshiaki Sone, Yosuke Kusano

**Affiliations:** 1 Department of Anthropology, The Ohio State University, 4034 Smith Laboratory, 174 W. 18th Avenue, Columbus, OH (43210-1106), USA; 2 Department of Creative Design, Tohoku Institute of Technology, 6 Futatsuzawa, Taihaku-ku, Sendai-shi, Sendai, Miyagi Prefecture, 982-8588, Japan; 3 Graduate School of Biomedical Sciences, Department of Public Health, School of Medicine, Nagasaki University, 1-12-4 Sakamoto, Nagasaki, 852-8523, Japan; 4 Department of Food and Nutrition, Osaka City University, 3-138, Sugimoto 3-Chome Sumiyoshi, Osaka, 558-8585, Japan; 5 Department of Community Development, Nagasaki Wesleyan University, 1212-1 Nishi-Eida-machi, Isahaya-shi, Nagasaki Prefecture, Isahaya, 854-0082, Japan

**Keywords:** Senescence, Aging, Functional loss, Activities of daily living, Asian, Disability

## Abstract

**Background:**

Between July and September 2005, a preliminary sampling of the elderly population of Hizen-Oshima Island, Nagasaki Prefecture, Japan was conducted by the local hospital’s nursing staff.

**Results:**

Reported here are preliminary results from this sample of 27 individuals with an average age of 71 years. Their ages ranged from 51 to 82 years, with a standard deviation (sd) of 7.4 years. In total, 33 aspects of physical and physiological variation were assessed on these 15 women and 12 men. As expected from previous studies of Japanese elders, our sample shows slightly elevated average blood pressure (142/81 mmHg, sd 16/10), but they are relatively lean (waist/hip = .9: sd 0.06) when compared to European or American standards. However, their average total cholesterol (TC = 210 mg/dl, sd = 42.8) is high compared to standards, as is their high-density lipoprotein cholesterol (HDLc = 55.4 mg/dl, sd = 15.1). Means, standard deviations (sd), ranges and upper bounds for quartile cut-points for all 10 variables used in the calculation of allostatic load (AL) were assessed. The overall average estimate for AL in this sample is 3.1 (sd = 1.58) and ranges from 1 to 7.

**Conclusion:**

AL shows variability across men and women, has little correlation with age, and is associated with physiological variation in blood glucose, dopamine and uric acid.

## Introduction

Among most populations today, more people are surviving past their sixth decades of life than ever before, emphasizing the need for understanding human physiological variation in senescence, aging, and degenerative diseases. Still most of our current knowledge of senescent processes comes from Western, mainly US and European, samples. As we no longer accept the view that most physiological decrements occurring with age are “normal” [1-3], we also must move beyond Western-centric understandings of senescent processes. Aging individuals must adapt to stresses imposed not only by their current environments and cultures, but also by their own survival and the ever-changing environments they encounter [2,4]. Among Western samples, loss of homeostasis secondary to altered DNA and protein expression, cellular dysfunction and ultimately organ-system failure are associated with decrements in physiological and psychosocial adaptability and life span [1,2,5-8].

Recent research on Western elders has led to new measurements for assessing functional declines as aspects of biophysical, physiological and psychosocial senescence; among these are allostatic load (AL) and frailty [3,8-15]. Neither the applicability of AL and frailty indices to non-Western populations nor the degree to which their associations with detrimental outcomes vary across different populations and cultural backgrounds have been widely assessed [3,8,16]. Secondary to culture, environment, and genetic backgrounds, variability in aging and senescence across populations should produce differences not only in frailty and AL but also in their associations with age, morbidity and mortality. Examined here is the degree to which an AL scale developed on US samples is associated with age and sex, usefully predicts morbidity (blood glucose, disability), and is associated with variation in physiological function (blood proteins and components) among Japanese elders. We examine the degree to which AL is associated with physiological function and differences among older Japanese men and women living on Hizen-Oshima Island, Nagasaki Prefecture in 2005. In addition, we assess the degree to which these factors and their associations with AL are influenced by sex and age in this elderly sample of Japanese citizens.

## Background

AL was designed to assess the cumulative effects of stress and stress responses on the soma. As originally developed, AL was assessed using a composite score of systolic and diastolic blood pressure, total and HDL-cholesterol, waist/hip ratio, glycated hemoglobin (Hb), serum dihydroepiandosterone-sulfate (DHEA-s), and urinary cortisol, noradrenaline and adrenaline (see Table 1; [8,10-13]). AL is an additive measure, determined by scoring its components as 1 in the highest quartile of risk (in the original formulation, the fourth for all but HDL-cholesterol and DHEA-s, for which highest risk is in the lowest quartile) and summing across measures (Table 1). Among US elders, AL correlates with age and predicts future dysfunction and mortality. Assessments often include other possible components of AL: for example C-reactive protein (CRP), glutathione, superoxide dismutase or pyruvate kinase [8]. AL appears to be associated with sarcopenia and reduced energy expenditure, reduced nutrition, weight loss, weakness, exhaustion, low physical ability and ultimately hospitalization and death. Given that several of the measures used to calculate AL also are included in the definition of Syndrome X, it could be suggested that AL is just another way of assessing Syndrome X. Elsewhere, we have shown that AL is not necessarily correlated with other aspects of glucose metabolism, such as blood glucose when glycated Hb and glucose are included or dropped from assessments of AL [3].

**Table 1:** The components of allostatic load after McEwen (1999)

**Secondary mediators of stress**	**Primary mediators of stress**
Systolic and diastolic blood pressure	Serum dihydroepiandosterone – sulfate
Waist/hip ratio	Overnight urinary cortisol, adrenaline, noradrenaline
HDL-cholesterol and total-cholesterol	
Glycated hemoglobin	

### Study location

A Japanese sample is ideal for this study: they are among the longest-lived people worldwide, are relatively homogeneous genetically, and persons residing in different regions of Japan follow different life styles. The population of Japan has aged rapidly in the late 20^th^ and early 21^st^ centuries, partly due to low and declining birth rates and partly due to improved survival at all ages, but particularly among those aged 65 years and older. This increase in older residents has led to increased needs for healthcare and pensions, but these elders may also show improved active life spans and health spans. Today about one-fifth of the Japanese population is aged 65 years and older; by 2020 this percent may rise to one-fourth of the 127 million total population wherein 25.6 million people currently are aged 65 years and older [17]. Of these, 25,000 are already centenarians and life expectancy was already 85.6 years among women in 2005 and 78.6 years among men. Given their increasing numbers, finding a simple method for predicting health outcomes and survival of elders will aid in focusing healthcare interventions and target those in most need for additional care.

### Hypotheses

Hypotheses tested here are: 1) AL differs between elderly Japanese men and women. 2) Higher AL is associated with older ages, greater morbidity and more extreme values of physiological factors observed in blood samples indicating poorer health. 3) Poorer responses to an Index of Competence designed by the Tokyo Metropolitan Institute of Gerontology are associated with higher AL.

### Population

At the time of this survey in 2005, the total population of Hizen-Oshima Island was 5,792 persons [17]. Of these, 2,917 were aged 50+ years, 1,523 were aged 65+ years, 733 were aged 75+ and 60 were over 90 years of age [17]. Until recent decades, a majority of residents practiced at least some traditional agriculture and fishing for their sustenance; however, Hizen-Oshima Island also was a major source of coal throughout the 19^th^ and 20^th^ centuries. During this period, large villages developed on the west side of the island facing Nagasaki. Additionally, 100's of persons migrated into this area from both the main island and other areas of Hizen-Oshima Island to work in the coal mines and provide needed services to miners. In the mid-20^th^ century, coal mining was abandoned and the port converted to use for ship building. Today, the shipyard continues to provide wage labor and economic stability to the western coast. Although the eastern coast has remained more traditional, prosperity and non-local foods and customs have permeated the entire island. This process accelerated during the late-20^th^ century following construction of a bridge linking the northwest coast of Hizen-Oshima Island to the main island.

## Methods

### Sample

Between July and September 2005, a preliminary sampling of the elderly population of Hizen-Oshima Island, Nagasaki Prefecture, Japan was conducted by the local hospital’s nursing staff under the direction of collaborating physicians, as part of the Japan Society for the Promotion of Science funded project, “Allostatic Load Among Hizen-Oshima Island Residents.” Hizen-Oshima residents receive health care at a single centrally located hospital, providing a single source for medical diagnoses and a single location to conduct medical exams. Dr. Y. Kusano hired nurses at Hizen-Oshima Island Medical Center to aid in biomedical protocols (blood draws, overnight urine collections and assessments of blood pressure). Each participant responded to a questionnaire including current perceptions of their health, previous health conditions, their current and past activity levels and the TMIG Index of Competence (Tokyo Metropolitan Institute of Gerontology Index of Competence). The TMIG index totals 13 questions about physical and social competency divided into 3 domains: Instrumental Self-Maintenance – 5, Intellectual Activity – 4, and Social Role – 4. Positive answers (ability to complete a task) are scored as 1; negative answers (inability to complete) are scored as 0. Scores are then summed similar to how AL is determined, except that higher TMIG scores indicate better function and less disability. Participants were recruited at their annual government sponsored physical examination through public health centers. Details and significance of the project, as well as the voluntary nature of participation were explained at the point of contact and all study protocols were incorporated into annual physical examination. Thus, as part of this study, each participant was provided additional testing of their blood chemistries, lipids and other medically important assessments. As hospital records, data generated by this project respected individual privacy and autonomy of participants to the highest degree possible, while also being available for clinical staff to use in treatment and diagnoses. Prior to being measured or completing any study protocols, participants provided informed consent according to protocols established at the Graduate School of Human Life Science, Department of Interdisciplinary Studies for Advanced Aged Society, Osaka City University.

Reported here are preliminary results from a random sample of 27 individuals with an average age of 71 years (Table 2). Ages ranged from 52 to 89 years, with an sd of 7.4 years (Table 3). Table 3 also provides descriptive statistics for variables examined for associations with allostatic load. In total, 33 aspects of physical and physiological variation were assessed for these 15 women and 12 men. Although this is a rather small sample of the total population aged 50+ years residing on the island, it does include about 0.9% of these and likely is sufficient to provide preliminary estimates of trends to be expected in associations of allostatic load with physiological outcomes among elderly residents of Hizen-Oshima Island. For one, residents maintain relatively similar lifestyles and are exposed to similar stressors and risk factors. Second, we have sampled randomly within a restricted age range of individuals who experienced similar historical events. Finally, participants came from all areas of Hizen-Oshima Island and do not represent a single local enclave.

**Table 2:** Descriptive statistics and cut-points for estimating allostatic load of elderly Hizen-Oshima Island residents

**Variable**	**Mean (sd)**	**Range**	**1**^**st**^**Q**	**2**^**nd**^**Q**	**3**^**rd**^**Q**
SBP	142 (16.4)	106 to 172	134	140	155
DBP	81 (10.4)	60 to 94	70	80	90
W/H	0.9 (.065)	0.74 to 0.97	0.80	0.87	0.89
HDLc	55.4 (15.1)	32 to 84	44	50	60
TOTc	210 (42.8)	142 to 315	182	203	238
Hb-g	5.4 (0.30)	4.9 to 6.2	5.2	5.3	5.5
DHEA	669 (366.6)	89 to 1580	408	585	854
CORT	15.5 (7.79)	2.8 to 30.1	9.1	14.5	22.6
AD	6.8 (6.41)	1 to 31	3.0	5.0	7.8
NAD	76.6 (44.2)	23 to 166	44	64	108

* Quartile cut-point for HDL-c and DHEA is the upper bound of the 1^st^. Abbreviations: SBP/DBP systolic/diastolic blood pressures, W/H waist: hip ratio, TOTc/HDLc total/high density cholesterol, Hbg glycated hemoglobin. DHEA Serum, dihydroepiandosterone – sulfate; CORT/AD/NAD, cortisol, adrenaline, noradrenaline.

**Table 3:** Descriptive statistics and ranges for dependent variables of elderly Hizen-Oshima Island residents

**Variable**		**Mean (sd)**	**Range**
AGE	(years)	70.8 (7.4)	51 to 82
WBC	(count/mm^3^)	5,834 (1,516)	3,500 to 9,700
GOT	(IU/L)	25.6 (6.7)	13 to 43
GPT	(IU/L)	20.0 (8.8)	8 to 42
Dopamine	(ng/dl)	747 (752)	139 to 3,490
Uric Acid	(ng/dl)	5.1 (1.2)	3.1 to 7.2
Glucose	(mg/dl)	116.8 (18.7)	94 to 174

### Statistical analysis

Means, standard deviations (sd), ranges and quartile cut-points were calculated for all variables used to estimate allostatic load (Table 2). As previously described, observations falling in the highest risk quartile were scored one and then summed to determine an allostatic load score. Differences between older and younger participants and between men and women were estimated and examined for statistical significance using standard t-tests comparing means. In these analyses, the indicator variable for sex was coded as 0 for women and 1 for men. Thus, results for sex differences reported as “significantly associated” indicate that men were higher and “significantly negatively associated” will indicate that women have a higher mean value for that measure. Similarly, main associations of AL with physiological variables were estimated using t-tests based upon a general liner model. Based upon observed univariate associations of AL, multiple regression models were used to determine independent effects of AL on several physiological measures controlling for age and sex.

## Results

Slightly elevated average blood pressure (142/81 mmHg, sd 16/10) is observed in this sample of Japanese elders (Table 2) compared to the United States (136/74 mmHg), but not European standards (150/85 mmHg at ages 65 to 69 years; see [18]). A relatively lean body habitus (waist/hip = 0.9: sd 0.06) also is observed in this sample compared to European men aged 55 to 64 years old (range 0.89 to 1.01), but not women (0.79 to 0.82; see [19]). Average total cholesterol (TC = 210 mg/dl, sd = 42.8) is rather high compared to international standards wherein less than 200 mg/dl is considered desirable. Conversely, high-density lipoprotein cholesterol (HDLc = 55.4 mg/dl, sd = 15.1) in this sample (Table 2) is above the 50 mg/dl determined as the international standard for healthy HDL-cholesterol; the ratio of total cholesterol to HDL-cholesterol of 3.8 in this sample is at the level determined as low risk (3.8 to 4.0 mg/dl) by international standards. Means, standard deviations (sd), ranges, and upper bounds for quartile cut-points for all 10 variables used in the calculation of allostatic load (AL) are listed in Table 2.

The overall average estimate for AL in this sample is 3.1 (sd = 1.58) and ranges from 1 to 7. There is a slight, non-significant tendency for estimated AL to be higher among those of younger age (3.4, standard error (se) = 0.457, *P* = 0.378) when the sample is divided into higher (2.9, se = 0.61) and lower age classes based upon the mean age of 71 years. However, age explains only about 3% of the variation in AL. Using regression, no significant linear or quadratic association of AL with age was observed (*P* >0.35 for both comparisons). Women showed lower AL on average (2.6, se = 0.37) than did men (3.7, se = 0.60). This difference was of borderline statistical significance (*P* = .088), explaining about 12% of the total variance in AL.

In this Hizen-Oshima Island sample, estimated AL is poorly predictive of blood glucose mg/dl (Table 3 includes means, standard deviations, ranges for outcome variables). A single unit increase in AL is associated with a 2.5 mg/dl decrease in blood glucose in this sample (se = 2.31, *P* = 0.30). AL only explains about 4% of the total variance in blood glucose, although glycated hemoglobin is included in the estimate of AL. Data from a number of studies also suggest that AL is associated with both physical and physiological dimensions of disability and frailty. In this sample, we collected data on ADLs (Activities of Daily Living) as indicated by an Index of Competence designed by the Tokyo Metropolitan Institute of Gerontology. Little variation in the Instrumental scale was observed (range 4 to 5, mean 4.89, sd = 0.32). Greater variation was observed in the other two sub-scales: Intellectual – range 0 to 4, mean = 3.04, sd = 1.13; Social - range 1 to 4, mean 3.41, sd 0.97. AL showed no significant associations with the total or any of the three sub-scales (*P* >0.40 for all four comparisons).

Because AL assesses stress from physiological arousal (hormones), subsequent poor health outcomes, and physical alterations (anthropometrics, lipids), AL was examined as a mediator of the psycho-physiologically active neurotransmitter, dopamine (mg/dl), a systemic antioxidant, uric acid (mg/dl), enzymes associated with liver function (GOT - glutamic oxaloacetic transaminase and GPT – glutamic pyruvic transaminase), and aspects of immune (WBC – white blood cells) and kidney function (creatine). In this sample, the strongest association of AL with any aspect of physiology is with the neurotransmitter dopamine, which averages about 747 ng/ml (sd = 752) (Table 4). For every one-unit increase in AL, dopamine increases about 184 ng/ml of blood (se = 87.9 ng/ml, *P* = 0.05) (Table 4), explaining about 15% of the total variation in dopamine for this sample. Similarly, AL is relatively strongly associated with uric acid mg/dl (mean 5.1, sd = 1.2) (Table 3), explaining about 12% of the total variation in uric acid levels for this sample (se = 0.15, *P* = 0.07). For every unit increase in AL, uric acid increases 0.274 ng/dl (Table 4). When age, sex and AL are jointly entered into multiple regression, AL remains positively associated with GPT (*P* = 0.021), blood sugar (*P* = 0.053), serum dopamine (*P* = 0.011), and WBC counts (*P* = 0.103), independent of sex and age (Table 4). Associations with blood pressure, blood sugar, noradrenalin, cortisol, and DHEA-s are expected, as these variables were included in the construction of AL. However, associations with dopamine (a neurotransmitter), uric acid (an antioxidant), GPT (a liver enzyme), and WBC counts (an indicator of inflammation), all aspects of physiology reflective of frailty, are not due to the construction of AL.

**Table 4:** Bivariate and multivariate associations of allostatic load with physiology among elderly Hizen-Oshima Island residents

**BIVARIATE ANALYSES**					
**Independent variables**	**Dependent variable**	**Regression coefficient**	**SE**	***P***	**R**^**2**^
AL	Glucose	- 2.47	2.31	0.30	0.04
AL	Dopamine	184.4	87.9	0.05	0.15
AL	Uric acid	0.274	0.15	0.07	0.12
AL	White blood cells	349.3	179.1	0.06	0.13
AL	GPT	1.86	1.05	0.09	0.11
AL	GOT	−0.292	0.88	0.74	0.04
Age	AL	−0.550	0.61	0.38	0.03
Sex (male = 1)	AL	1.075	0.60	0.09	0.12
MULTIVARIATE MODELS CONTROLLING FOR AGE AND SEX					
Age	Glucose	- 0.006	0.48	0.99	
Sex	21.42	7.54	0.01		
AL		- 4.79	2.34	0.05	0.32
Age	Dopamine	15.1	19.6	0.89	
Sex		- 645.2	309.6	0.05	
AL		265.1	96.1	0.01	0.29
Age	Uric acid	−0.036	0.03	0.23	
Sex		1.452	0.47	0.005	
AL		0.080	0.14	0.45	0.38
Age	White blood cells	- 463.2	597.0	0.45	
Sex		32.9	37.8	0.39	
AL		315.1	185.4	0.10	0.12
Age	GPT	−0.136	0.23	0.55	
Sex		- 7.04	3.57	0.06	
AL		2.75	1.11	0.02	0.30
Age	GOT	−0.247	0.18	0.19	
Sex		- 6.00	2.89	0.05	
AL		0.189	0.90	0.84	0.29

(The first eight rows are bivariate results, while the lower rows present the same analyses including the effects of age and sex).

## Discussion

In this sample, AL is associated independently with physiological variation, including dopamine, uric acid, GPT, and WBC counts, and shows variability across men and women, but is only poorly correlated with age and serum glucose. Given the restricted age range in this sample (51 to 82 years) and its small size, it is not surprising that little association of AL with age was observed. Additionally, reported associations are based upon a slightly higher alpha value (0.10) than the standard 0.05. However, this seems reasonable given the exploratory nature of this study and the small sample size obtained for this pilot study of methods and techniques. It is interesting that in this older sample, AL shows significant associations with a neurotransmitter, a liver enzyme, an antioxidant, and an inflammatory marker, measures representing a variety of somatic functions widely reported to be altered with senescence/aging and associated with cognitive function and frailty. Inflammation has been described as “the fire of frailty” by Walston (2005) and colleagues (Fried *et al.* 2004) [9,15]. The observed borderline association of WBCs with AL in this small sample suggests a connection between these two measures of function in elders and processes of senescence. Similarly, the association between AL and uric acid suggests a connection between reactive oxygen species and higher AL. Although these later are conjectures from the observed data, they do suggest that further exploration of how AL is associated with physiological function in older Japanese adults is warranted. No significant associations of AL with the TMIG index or any of its three subscales were observed. This suggests that although AL influences physiological variation among elderly Japanese, these effects are not altering elders’ somatic competence.

Data reported here also indicate that AL may be usefully applied across cultures to assess alterations in physiological risks and differences across subgroups within populations that may be associated with senescence/aging. Most importantly, we observed that AL in this sample is most closely associated with two aspects of physiology, serum dopamine and uric acid concentrations that have been shown to decline with age in other samples, see [7]. Additionally, older Hizen-Oshima Island women show lower AL than do older men, as would be expected given that women commonly live longer. However, in other samples, women tend to report greater disability at older ages than do men [20]. Finally, unexpected associations of AL with enzyme function and inflammation require further investigation.

## Abbreviations

ADL: Activities of daily living; AL: Allostatic Load; CRP: C-reactive protein; DHEA: Dihydroepiandosterone-sulfate; GOT: Glutamic ocaloacetic transaminase; GPT: Glutamic pyruvic transaminase; Hb: Hemoglobin; HDLc: High-density lipoprotein cholesterol; Sd: Standard deviations; Se: Standard error; TC: Total cholesterol; TMIG: Tokyo Metropolitan Institute of Gerontology; WBC: White blood cells.

## Competing interests

The authors declare that they have no competing interests.

## Authors’ contributions

DEC, participated in study design and coordination, performed the statistical analysis, and drafted the manuscript. HH, participated in study design, helped acquire funding from the Japan Society for the Promotion of Science. KA, participated in study design, helped coordinate research activities. TM, participated in study design, helped coordinate research activities. AA, performed statistical analysis. YK, participated in study design, helped coordinate research activities, involved in drafting the manuscript. YS, participated in study design and coordination, involved in drafting the manuscript, helped acquire funding from Japan Society for the Promotion of Science. All authors read and approved the final manuscript.
